# Bridging the Data Divide in Nevada: A Repeated Cross-Sectional Study of Birth Certificate and Medicaid Billing Discrepancies in Gestational Substance Exposure

**DOI:** 10.3390/healthcare14020238

**Published:** 2026-01-18

**Authors:** Kyra Morgan, Kavita Batra, Stephanie Woodard, Erika Ryst, Paul Devereux, Wei Yang

**Affiliations:** 1 Interdisciplinary Environmental Sciences and Health PhD Program, University of Nevada, Reno, NV 89557, USA; weiyang@unr.edu; 2 Division of Child and Family Services, Nevada Department of Human Services, Carson City, NV 89706, USA; 3 Department of Medical Education, Kirk Kerkorian School of Medicine at UNLV, University of Nevada, Las Vegas, NV 89102, USA; 4 Office of Research, Kirk Kerkorian School of Medicine at UNLV, University of Nevada, Las Vegas, NV 89102, USA; 5 School of Public Health, University of Nevada, Reno, NV 89557, USA; stephaniewoodard@unr.edu (S.W.); devereux@unr.edu (P.D.); 6 College of Education and Human Development, University of Nevada, Reno, NV 89557, USA; eryst@med.unr.edu; 7 Nevada Center for Excellence in Disabilities, University of Nevada, Reno, NV 89557, USA; 8 Interdisciplinary Social Psychology PhD Program, University of Nevada, Reno, NV 89557, USA

**Keywords:** gestational exposure to substances (GES), maternal substance use, prenatal substance exposure, multi-level modeling, Medicaid-covered births, reporting discordance, integrated surveillance, Nevada

## Abstract

**Background/Objectives**: Gestational exposure to substances (GES) is associated with adverse developmental outcomes. Early identification is limited by reliance on self-reported data. This study assessed the incidence and predictors of discordance in GES reporting between birth certificates and Medicaid claims among Medicaid-covered births in Nevada from 2022 to 2024. **Methods**: A statewide, hospital-clustered, cross-sectional analysis was conducted using linked Medicaid billing and birth record data. Discordance was defined as GES identified in one source but not the other. Incidence per 1000 live births was stratified by demographic characteristics. Multilevel logistic regression assessed patient- and hospital-level predictors, with random hospital intercepts. **Results**: Among 50,394 live births, the discordance rate was 95.09 per 1000 (95% Confidence Interval: 92.5–97.7). Substantial disparities were observed by race/ethnicity, socioeconomic status, and geography, with higher discordance among White non-Hispanic mothers, those residing in rural or frontier counties, and individuals with lower educational attainment or living in lower-income areas. Modest but meaningful variation was also observed across hospitals, including differences by hospital size and teaching or research status. **Conclusions:** Findings highlight substantial discordance in GES reporting and underscore the limitations of single-source surveillance. Findings also have clear policy relevance, indicating that improved cross-system data integration would strengthen statewide surveillance, enhance early detection, and support more equitable resource allocation and intervention strategies.

## 1. Introduction

Gestational exposure to substances (GES) represents a persistent and under-recognized public health and surveillance challenge in the United States. Accurate population-level identification of GES is essential for monitoring trends, allocating resources, and ensuring timely access to prevention and early intervention services. However, current surveillance systems for GES rely on data sources that vary widely in completeness, accuracy, and susceptibility to reporting bias. As a result, reliance on any single data source may substantially underestimate the true prevalence of GES, limiting the ability of public health systems to respond effectively. These challenges are particularly salient in states such as Nevada, where diverse populations, geographic heterogeneity, and fragmented health data systems may further contribute to under-identification and misclassification of GES.

One important challenge in GES surveillance is the discordance between different data sources. Discordance may reflect identification error or differences in clinical documentation. It represents a critical limitation of existing surveillance approaches with meaningful implications for public health planning, resource allocation, and policy development. Under-identification or misclassification of GES can lead to an underestimation of population-level prevalence, limit timely intervention for affected infants, and obscure disparities across demographic groups and healthcare settings.

The public health importance of accurate GES surveillance is underscored by the substantial and well-documented consequences of prenatal substance exposure. GES can significantly impact long-term health and overall quality of life. For example, prenatal opioid exposure has been associated with neonatal abstinence syndrome, prenatal alcohol exposure with fetal alcohol spectrum disorders, and prenatal cannabis or stimulant exposure with cognitive and behavioral difficulties in childhood. It is associated with a range of adverse outcomes, including developmental delays, learning disabilities, and speech and language disorders that are often identified in early childhood and linked to diminished academic performance in later years, including high school [1,2,3,4]. Despite these well-established associations, current strategies for early identification of GES are inadequate, limiting critical opportunities for timely intervention that could mitigate adverse developmental trajectories throughout childhood and adolescence [5,6,7].

In Nevada, surveillance and identification challenges are further shaped by the regulatory and practice context surrounding prenatal substance exposure. Existing intervention efforts for GES are primarily directed toward infants who present with observable symptoms at birth, such as those diagnosed with Neonatal Abstinence Syndrome (NAS), Neonatal Opioid Withdrawal Syndrome (NOWS), or Fetal Alcohol Syndrome (FAS). However, children affected by GES who do not receive a formal diagnosis at birth often face significant barriers to care. This disparity is compounded by limited access to specialized providers, many of whom have extensive waitlists, delaying critical early interventions that are essential for improving developmental outcomes. These challenges are further exacerbated by concerns among pregnant individuals regarding mandatory substance use reporting, which may deter disclosure due to fears of child protective services involvement or other legal repercussions. In Nevada, healthcare providers are required to notify child welfare authorities when an infant is identified as substance-affected, including cases involving prenatal substance exposure, withdrawal symptoms, or fetal alcohol spectrum disorders. Although such notifications do not automatically result in child welfare investigations and are intended to support service coordination through Comprehensive Addiction and Recovery Act (CARA) Plans of Care, awareness of these requirements may influence both patient disclosure and provider documentation practices, contributing to discordance across data sources. Such fears have been recognized in clinical guidance, which emphasizes that punitive approaches can undermine care access and compromise maternal and infant health outcomes [8]. National clinical frameworks, including the AIM Patient Safety Bundle for Substance Use in Pregnancy, similarly underscore the need for non-punitive approaches to identification and care, emphasizing coordinated systems that improve maternal–infant outcomes [9].

A recent report from the Nevada Health Authority, Office of Analytics, found that approximately 12% of births to Nevada residents were GES in any given year from 2018 to 2020 [10]. The report incorporated several data sources including vital records, Medicaid claims, hospital billing data, and CARA plans of safe care. It found that many of these exposures were not recorded on the birth certificate or within the state’s comprehensive vital records database, which is widely regarded as the primary repository for birth-related statistics [11,12]. This underreporting on the birth certificate is likely due to the primary reliance on self-reported data for substance-use, which is vulnerable to reporting and social desirability bias [8,13,14].

Various studies have attempted to quantify discordance between self-reported GES and GES identified through other means, including urine screenings [15,16], umbilical cord sampling [17], medical records [18] and other health databases [19]. For example, a 2019 study by Metz et al. compared self-reported prenatal marijuana use with umbilical cord sampling among births over a 12-day period at two urban medical centers in Colorado and identified a 4-to-8-fold increase in identification of prenatal marijuana use among those sampled when compared to self-reporting [17]. However, the existing literature is limited in several ways. Many studies focus narrowly on specific substances [15,16,17,18] or selected populations [15,16,17], limiting the generalizability of their findings to Nevada’s population. Other studies rely on aggregate data and do not link individual-level records across data sources [16,18,19]. Additionally, several studies focus more narrowly on understanding specific substance-use-related outcomes, like NAS, rather than overall GES [20,21,22].

Population-level research on the comprehensive identification of GES that is generalizable is lacking, and this paper is intended to fill these gaps. This study contributes to the existing literature in three keys ways. First, it comprehensively quantifies the most recent and precise incidence of discordance in reporting of GES, utilizing linked data from two large population-based databases. Secondly, it is specific to Nevada’s population characteristics and includes jurisdictional comparisons at the regional level. This is important because previous literature has demonstrated significant variations in substance use disorder generally as well as outcomes related to GES, like NAS, at the state and regional levels [20,22,23,24]. Third, it evaluates both patient-level and hospital-level predictors of discordance in reporting of GES, which can help local public health and social service programs to target interventions at this level of granularity [24]. This study also serves as a guide for future research in the utility of administrative claims data for identification of GES and quantifies the measurable gaps in self-reported GES in specific demographic subgroups. It provides a framework that other states or regional health authorities, medical providers, researchers, and other stakeholders can follow to re-create similar analyses for their respective populations.

Improved identification of infants with GES, including those without obvious symptoms at birth or maternal disclosure, would enable public health officials to better allocate resources to support affected children and families [13], via expedited developmental screening and early intervention, thereby minimizing adverse outcomes. Despite this need, we are not aware of prior studies that assess individual-level discordance in GES reporting across linked administrative data sources while simultaneously accounting for hospital-level clustering. Therefore, the aim of this study is to quantify the incidence of discordance in GES reporting between Nevada birth certificates and Medicaid claims and to identify patient- and hospital-level predictors of this discordance using linked, population-based administrative data.

## 2. Methods

### 2.1. Study Design and Data Sources

This retrospective, observational study utilized a repeated cross-sectional study design. A statewide, hospital-clustered analysis was employed, using multiple cross-sectional data sources extracted from state and federal administrative databases. Data were sourced from the Nevada Health Authority, Office of Analytics, the American Community Survey 2023 5-year estimates, and the Nevada Hospital Association [25,26,27].

### 2.2. Ethical Considerations

This study (Project ID: 2345576-1) was reviewed and approved by the University of Nevada, Reno Institutional Review Board (IRB) on 15 July 2025. The IRB determined that the study met the criteria for ethical conduct of research involving human subjects in accordance with federal regulations and institutional policy. All data were de-identified prior to analysis, and no individually identifiable information was used or disclosed.

### 2.3. Sample Selection

**Figure 1:**
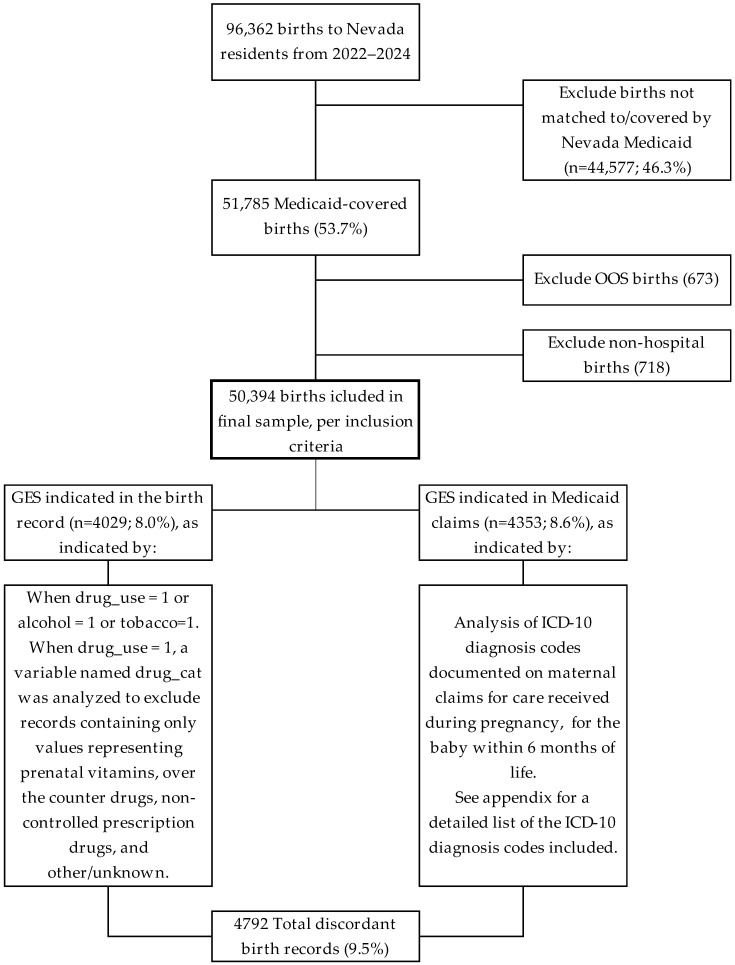
Inclusion/exclusion criteria diagram. Detailed inclusion and exclusion criteria are described in Section 2.3. OOS = Out of State.

The study population includes all babies born to Nevada residents, in Nevada hospitals, between 2022 and 2024 where the birth was paid for by Nevada Medicaid and corresponding records existed in both datasets for comparison of discordance (N = 50,394; Figure 1; Table 1). Multiple births are included in the analysis with no analytical adjustments (n = 537; 1.07% of birth records). Non-hospital births were excluded due to inconsistent documentation and because our models assume clustering within hospitals.

### 2.4. Statistical Analysis

Nevada Medicaid claims data were evaluated for the presence of GES via an analysis of ICD-10 diagnosis codes documented on maternal claims for care received during pregnancy, as well as claims billed for the baby within 6 months of life. See Appendix A for a detailed list of the ICD-10 codes included. Birth certificate data corresponding to these deliveries were then evaluated for the presence of GES via an analysis of three existing dichotomous, primarily self-reported variables indicating use of GES (drug_use = 1, alcohol = 1, or tobacco = 1). When GES was identified on the birth record via an indicator value of drug_use = 1, a field named ‘drug_cat’ was analyzed to exclude records containing only the values related to prenatal vitamins, over the counter drugs, non-controlled prescription drugs, and other/unknown. Each birth was then assigned three new dichotomous variables:

- a dichotomous variable indicating GES based on Medicaid claims analysis (1 = GES, 0 = no GES);
- a dichotomous variable indicating GES based on birth certificate analysis (1 = GES, 0 = no GES); and
- a dichotomous variable indicating discordance between the two as identified by having GES indicated on one source but not the other (DISCORDANCE: 1 = discordant, 0 = concordant). This is the primary outcome variable of interest (Table 2).

Incidence rates for discordance per 1000 live births were calculated by dividing the number of GES discordant birth records by the total number of live birth records and multiplying that result by 1000. Overall rates of discordance are presented by demographic variables including mother’s age, race/ethnicity, county of residence, educational attainment, and resident zip code median household income. Corresponding 95% Wald confidence intervals, which are appropriate for large sample sizes due to central limit theorem, were derived using normal approximation methods under the assumption of binomial distribution and implemented via SAS. Wald confidence intervals were used due to their computational simplicity and acceptable performance with large sample sizes and moderate proportion values [28].

**Table 1:** Patient-level characteristics among birth records.

	Number of Birth Records (n = 50,394)			
	2022	2023	2024	2022–2024 Total
Statewide	17,221	16,523	16,650	50,394
Age				
<18	266	288	277	831
18–29	10,394	9735	9636	29,765
30–39	6065	5930	6165	18,160
40+	496	570	586	1652
Race/Ethnicity				
White non-Hispanic	4453	4114	4093	12,660
Black non-Hispanic	3425	3229	3138	9792
Hispanic	8015	7907	8176	24,098
Other non-Hispanic	1232	1154	1112	3498
Mother’s County of Residence				
Clark	13,911	13,219	13,274	40,404
Washoe	1988	2050	2081	6119
Rural/Frontier	1321	1251	1292	3864
Educational Attainment				
Less than High School	3516	3253	3266	10,035
High School or GED	7370	7204	7193	21,767
Some College	4415	3970	3885	12,270
Bachelors Degree	1024	955	1072	3051
Masters or Higher	255	223	213	691
Unknown	641	918	1021	2580
Zip Code Median Household Income				
Quartile 1 (≤USD 50,945)	4420	4328	4237	12,985
Quartile 2 (USD 50,946–USD 65,798)	4276	3987	4166	12,429
Quartile 3 (USD65,799–USD 85,801)	4408	4295	4236	12,939
Quartile 4 (≥USD 85,802)	4081	3879	3979	11,939

**Table 2:** Patient-level characteristics among discordant birth records.

	Number of Discordant Birth Records (n = 4792)			
	2022	2023	2024	2022–2024 Total
Statewide	1694	1578	1520	4792
Age				
<18	30	27	31	88
18–29	997	929	862	2788
30–39	615	584	578	1777
40+	52	38	49	139
Race/Ethnicity				
White non-Hispanic	746	628	612	1986
Black non-Hispanic	433	405	419	1257
Hispanic	408	426	393	1227
Other non-Hispanic	86	91	70	247
Mother’s County of Residence				
Clark	1183	1112	1116	3411
Washoe	282	261	211	754
Rural/Frontier	229	204	192	625
Educational Attainment				
Less than High School	410	377	369	1156
High School or GED	789	736	665	2190
Some College	357	325	314	996
Bachelors Degree	40	25	44	109
Masters or Higher	9	6	7	22
Unknown	89	109	121	319
Zip Code Median Household Income				
Quartile 1 (≤USD 50,945)	423	419	400	1242
Quartile 2 (USD 50,946–USD 65,798)	468	389	374	1231
Quartile 3 (USD 65,799–USD 85,801)	456	444	400	1300
Quartile 4 (≥USD 85,802)	339	321	343	1003

Multilevel logistic regression models were used to investigate potential predictors at both the patient and hospital level. Patient-level predictors for mother’s age, race/ethnicity, county of residence, educational attainment, and median household income were evaluated. Hospital-level predictors included hospital type (public vs. private), hospital academic status (teaching/research vs. non-teaching/research hospital), hospital location (rural vs. urban), and hospital bed size based on quartiles (≤60, 61–199, 200–393, 394+). For all categorical variables included in the regression models, reference categories were selected to facilitate interpretation and provide meaningful comparisons. Where possible, the category with the largest sample size was chosen (e.g., Age: 18–29, County: Clark, Education: High School, Bed Size: 200–393). Race/Ethnicity was set to White non-Hispanic to maintain consistency with prior literature, and the Income reference category corresponds to Nevada’s median household income to provide a meaningful baseline for comparisons. The intraclass correlation coefficient (ICC) was calculated to measure hospital-level clustering. Generalized linear mixed models, implemented via the GLIMMIX procedure, were used to account for clustering of patients within hospitals. Gauss–Hermite Quadrature likelihood approximation was used to achieve model convergence.

Four models were considered for goodness of fit: a null model that did not consider any patient- or hospital-level predictors and only evaluated for the presence of discordance (Model 0); a null model that did not consider any patient- or hospital-level predictors but included hospital clusters as random intercepts (Model 1); a model that considered patient-level predictors as fixed effects and hospital clusters as random intercepts (Model 2); and a model that included both patient- and hospital-level fixed effects and hospital clusters as random intercepts (Model 3). Models were compared in a stepwise manner using Likelihood Ratio Tests (LRTs) for goodness of fit, as well as Akaike Information Criterion (AIC) and Bayesian Information Criterion (BIC) to account for the trade-off between model complexity and parsimony (Table 3). Multicollinearity was assessed using Variance Inflation Factors (VIFs), with values above 5 indicating potential concern. All predictors had VIFs below this threshold, suggesting multicollinearity was not an issue. To evaluate the robustness of findings to missing data, we conducted a sensitivity analysis by re-estimating the model after excluding records with missing values on any predictor variable. All statistical analyses were conducted using SAS software version 9.4 (SAS Institute Inc., Cary, NC, USA).

## 3. Results

### 3.1. Incidence of Discordance [Descriptive Statistics]

The overall rate of discordance was 95.09 per 1000 live births (95% CI 92.5, 97.7) during the study period (2022–2024) (Table 4). There were no significant differences in the unadjusted rate of discordance across years or based on mother’s age (Table 4).

Rates of discordance differed significantly among different racial and ethnic populations. White non-Hispanic mothers experienced the highest rate of discordance (156.87; 95% CI 150.5–163.2), followed by Black non-Hispanics (128.37; 96% CI 121.7–135.0). Hispanic and other non-Hispanic minority populations had the lowest rates of discordance at 50.92 (95% CI 48.1–53.7) and 70.61 (95% CI 62.1–79.1), respectively (Table 4). All racial and ethnic groups had consistent rates of discordance over the study period, with no significant increases or decreases across years.

Regional comparisons were conducted based on mother’s county of residence (Table 4). Rural and frontier Nevada observed the highest rates of discordance at 161.75 per 1000 live births (95% CI 150.1–173.4), followed by Washoe County (123.22; 95% CI 115.0–131.5) and Clark County (84.42; 95% CI 81.7–87.1). Washoe county observed a significantly lower rate of discordance in 2024 compared to prior years, while other regions had consistent rates from 2022 through 2024.

When considering birthing mother’s educational attainment, we found that discordance is negatively associated with increasing education (Table 4). Mothers with high school equivalency or less were more likely to have discordant records than mothers with some college. Similarly, mothers with only some college but without a degree were more likely to have discordant records than mothers with a bachelor’s degree or higher (bachelor’s degree: 35.73 (95% CI 29.1–42.3); master’s or higher: 31.84 (95% CI 18.7–44.9)). Discordance also varied by mother’s resident zip code median household income. Specifically, mothers residing in the upper quartile zip codes (≥USD 82,599) demonstrated lower rates of discordance (84.01; 95% CI 79.0–89.0) compared to all other quartiles.

### 3.2. Predictors

To test for predictors, we assume that births are nested within hospitals. We found that the model with the random hospital intercept (Model 1) is significantly better than the single-level model (Model 0). Model 1 estimated how much of the variability in discordance was due to differences between hospitals. From this, we determine that the predicted probability of discordance at an average hospital is about 13% and the variance of the random intercepts at the hospital level is 0.01063. Further, the ICC was 0.0412, demonstrating that 4.1% of the variability in discordance is due to differences at the hospital level. This indicates small but potentially meaningful clustering (Table 3).

As expected, when patient-level predictors were added to the model, it performed better than the null model based on LRT comparisons for goodness of fit (Table 3). Similarly, Model 3, which incorporated both hospital- and patient-level predictors, outperformed Model 2. This demonstrates that there is significant variation in discordance based on hospital clusters. More specifically, when considering hospital-level predicators, teaching/research status was a significant predictor, along with small hospital size.

### 3.3. Final Model

We identified significant fixed effects for mother’s age group, race/ethnicity, county of residence, education level, and resident zip code median income quartile as well as hospital teaching/research status and bed capacity. All independent variables were categorical and included in the model as dummy variables. To account for clustering of patients within hospitals, we included a random intercept for facility. This allowed the baseline odds of discordance to vary across facilities. The final model gives log-odds of DISCORDANCE (Y_ij_ = 1) for birth i in hospital cluster j as a function of fixed effects (predictors) and a random intercept for each hospital.

$$logit\left(P\left(Y_{ij}=1\right)\right)=\beta_{0}+\sum_{k}\beta_{k}X_{kij}+b_{j}$$

where:

$$Y_{ij}=The\;outcome,\;discordance,\;for\;birth\;i\;in\;hospital\;j$$

$$\beta_{0}=the\;overall\;intercept$$

$$\beta_{k}=the\;fixed\;effect\;coefficients\;for\;each\;level\;of\;the\;categorical\;predictors$$

$$X_{kij}=The\;set\;of\;dummy-coded\;fixed\;effect\;predictors$$

$$b_{j}=the\;random\;effect\;for\;hospital\;j,\;\mathrm{and}\;b_{j}\sim N\left(0,\sigma^{2}\right)$$

In terms of age, mothers aged 30–39 at time of birth had 12.6% higher odds (OR: 1.126; 95% CI: 1.054–1.203) of discordance compared to those aged 18–29 years (reference group) (Table 5). In terms of race/ethnicity, Black non-Hispanic mothers had almost 13% reduced odds of discordance (OR: 0.873; 95% CI 0.802–0.949), and Hispanic mothers had 73% reduced odds of discordance compared to their White non-Hispanic counterparts (reference) (OR: 0.268; 95% CI: 0.247–0.291) (Table 5). Mother’s educational attainment also played a significant role—those with less than a high school diploma had nearly 40% increased odds compared to high school graduates or equivalent (reference) (OR: 1.396; 95% CI 1.286–1.514), while those with higher education levels had reduced odds of discordance, with up to 72% reduced odds for mothers with a master’s degree or higher (Table 5). Finally, mother’s living in rural and frontier counties had 55% increased odds of discordance compared to Clark County (reference) (OR: 1.550; 95% CI 1.299–1.851). Those living in zip codes with median household income in the upper quartile saw more than a 20% reduction in odds of discordance compared to mothers living in zip codes in the 3rd median income quartile (reference) (OR: 0.797; 95% CI 0.727–0.874) (Table 5). Conversely, the odds of discordance for mothers residing in 1st quartile zip codes was 10% higher than the reference group.

Sensitivity analyses limited to complete cases (i.e., excluding records with missing predictor values) produced comparable estimates to the primary analysis (N = 47,730; 94.7% of the total study population); however, statistically significant differences emerged between Nevada’s two major urban regions (Washoe and Clark Counties) such that Washoe County was found to have nearly 35% higher odds of discordance compared to Clark County (reference) when records with any unknown predictor values were excluded (OR: 1.347; 95% CI 1.039–1.746).

In terms of hospital-level characteristics, teaching/research hospitals had 35.5% higher odds (OR: 1.3556; 95% CI: 1.084–1.694) of discordant reporting compared to non-teaching/research hospitals (reference group) (Table 6). Hospitals in the first size quartile, with less than or equal to 60 beds, also had 69.5% increased odds of discordance (OR: 1.695; 95% CI: 1.118–2.571) compared to the reference group (Table 6).

## 4. Discussion

This study aimed to quantify discordance in GES reporting between Nevada birth certificates and Medicaid claims and to identify patient- and hospital-level predictors of this discordance. Consistent with these objectives, we found substantial discordance between data sources, with discordance identified on 95.09 per 1000, or approximately 9.5%, of live births that occurred in Nevada from 2022 to 2024 where Medicaid was the payer. Despite significant differences among groups, discordance was high across the population. Further, when we consider the combined percentage of records identified with GES using either/or logic from both data sources, we quantify that 13.1% of the study population experienced GES. This yields increased identification of GES by 5.1 percentage points and demonstrates the value of utilizing medical claims data to identify GES. The results align with our expectations based on previous literature, which has demonstrated self-reported substance use may be underreported by anywhere from 7 to 24 percentage points compared to biometric testing [16,17,18,19,29]. These findings underscore the limitations of relying on a single data source for GES surveillance and demonstrate that linked administrative data can meaningfully improve population-level identification of GES.

GES indicated on birth certificates and Medicaid claims may partly reflect repeated verification across clinical encounters. However, the magnitude and consistency of discordance in our analyses indicate these differences are substantial. As shown in Figure 1, the number of GES-identified births captured in Medicaid claims (n = 4353; 8.6%) slightly exceeded the number identified through birth records (n = 4029; 8.0%). Although the primary focus of this study is discordance rather than prevalence estimation, this pattern is consistent with prior concerns that birth certificate fields relying on patient self-report or provider recognition alone systemically underestimate GES. Similar discrepancies between self-reported prenatal substance use and alternative data sources have been documented in prior studies using biological testing and medical records, which consistently demonstrate higher identification when self-report is supplemented [16,17,18,19,29].

Of key importance are maternal demographics. After controlling for other covariates, discordance was disproportionately observed among mothers aged 30 to 39 and mothers of White non-Hispanic racial and ethnic composition. Several mechanisms may contribute to these patterns. Enhanced clinical surveillance among younger and older mothers due to perceived obstetric or social risk increase GES identification in clinical settings [30]. At the same time, variation in the prevalence of substance use dependence and overdose-related hospitalizations among women of reproductive age in Nevada may differentially influence documentation practices across demographic groups [31]. In addition, stigma and fear of punitive consequences may also play a critical role. Prior research suggests that concerns about child protective services involvement can suppress disclosure of substance use during pregnancy, with effects varying by age, race/ethnicity, and prior experiences with the healthcare system [8,12,13,14]. Differences in trust, communication, and healthcare engagement across populations may therefore contribute to observed disparities in discordance.

In addition, our findings emphasize an association between discordance and mother’s socio-economic status. More educated mothers and mothers residing in zip codes with higher median income were less likely to have discordant records. One plausible explanation is that higher educational attainment may be associated with greater access to information about the risks of GES, which in turn may reduce the likelihood of exposure during pregnancy. Prior research has demonstrated lower rates of prenatal substance use among individuals with higher socioeconomic status, supporting plausibility of this interpretation [32,33]. Socioeconomic position may also influence the consistency of documentation through differences in healthcare engagement and communication. Individuals with higher education and income may be more likely to initiate prenatal care earlier, maintain continuity of care, and interact more frequently with healthcare providers, increasing opportunities for substance use screening and consistent documentation across clinical and administrative systems. Conversely, individuals in lower socioeconomic contexts may face greater concerns about stigma or punitive consequences related to substance use disclosure during pregnancy. Fear of involvement with child protective services or other legal repercussions has been shown to suppress disclosure, particularly among populations that have historically experienced heightened surveillance or negative interactions with healthcare and social service systems [8,13,14]. Together, differences in health literacy, prenatal care engagement, and stigma-related disclosure likely contribute to socioeconomic disparities in documentation consistency across data sources.

Rural and frontier Nevada observed higher rates of discordance when compared to Nevada’s two metropolitan counties. Prior literature has demonstrated increased rates of maternal opioid use and NAS among rural populations compared to their urban counterparts [21], suggesting that underlying variation in substance use patterns may contribute to differential discordance. This may reflect structural barriers to care, limited access to prenatal services, or differences in screening and documentation practices. Our study extends the literature by demonstrating that rural–urban differences are also evident in the discordance of GES reporting across administrative data sources, suggesting that geographic disparities may affect not only substance use patterns, but also surveillance quality. Lastly, the observed associations with teaching/research status and hospital bed size suggest that institutional factors, such as clinical protocols, staffing capacity, documentation practices, and training, may influence whether GES is consistently captured across data systems. These findings highlight hospitals as critical intervention points for improving GES surveillance, particularly through standardized screening, documentation workflows, and alignment between clinical and administrative records. These findings also have important implications for public health surveillance and policy, which are discussed in the following section.

### 4.1. Strengths

- Novel contribution: To our knowledge, this is the first study to quantify individual-level discordance in GES reporting across linked administrative data sources in Nevada, stratified by demographic and geographic characteristics, while accounting for hospital-level clustering.
- Large, population-based sample: This study uses statewide data covering Medicaid-financed births in Nevada from 2022 to 2024, representing over half of all births in the state and providing robust population-level estimates [34].
- Linked administrative datasets: By linking birth certificate records with Medicaid claims data at the individual level, this study overcomes limitations inherent to single-source surveillance and enables direct assessment of discordance in GES reporting.
- Multilevel analytic approach: The use of hospital-clustered multilevel logistic regression accounts for patient nesting within hospitals and allows examination of both patient- and hospital-level predictors of discordance.
- Replicability: The analytic approach provides a simple repeatable framework that relies on routinely collected administrative data and can be readily adapted by other states or jurisdictions with similar data infrastructure with a low-cost burden.

### 4.2. Limitations

- Cross-sectional design: The study captures exposure and outcome information at a single point in time, precluding causal inference and limiting assessment of temporal relationships or changes in reporting patterns over time.
- Reliance on administrative data: Both birth certificate and Medicaid claims data are subject to underreporting and misclassification. Substance use may only be documented in claims when it reaches a severity threshold requiring medical intervention, potentially leading to underestimation of GES in both data sources.
- Disclosure and documentation bias: Hesitancy to disclose substance use during pregnancy, which is often driven by concerns about reporting to child protective services or law enforcement, may suppress documentation [8,13,14], particularly for substances perceived as lower risk, such as cannabis.
- Residual confounding: Despite adjustment for multiple sociodemographic and hospital-level factors, unmeasured influences (e.g., provider practices, institutional policies, or regional reporting norms) may affect observed associations.
- Directionality of discordance: This study does not determine which data source more accurately captures true GES. Birth certificate data are subject to recall and social desirability bias, while claims data may reflect ascertainment bias due to coding practices. Future studies incorporating biological confirmation or medical chart review could help clarify the origins and direction of discordance.
- Generalizability: Although Medicaid covers more than 50% of births in Nevada [34], findings may not be fully generalizable to non-Medicaid populations or to other states with different healthcare systems or reporting practices.

## 5. Policy Implications

Findings from this study demonstrate that reliance on single-source data, specifically birth certificates alone, substantially underestimates the burden of GES and obscures disparities across populations, regions, and healthcare settings. Although self-report remains the recommended best practice for screening pregnant patients for substance use, particularly when conducted within a non-punitive clinical environment [9], its effectiveness depends heavily on patient comfort, perceived safety, and consistency of implementation across settings. In practice, these ideal conditions are not always met, resulting in under-disclosure and gaps in documentation, especially for substances perceived as lower risk. Policymakers should prioritize integration of Medicaid claims and vital records for routine cross-verification of GES to improve surveillance completeness and accuracy.

Furthermore, the severity, frequency, duration, and type of substance used all influence the likelihood that exposure will be identified, documented, and ultimately detected in administrative or vital records data. Recognizing these limitations, there is a need to shift identification efforts further upstream. Detecting substance use risk earlier in pregnancy—not solely at the time of delivery—would support opportunities to reduce exposure, engage patients in treatment, and ultimately decrease GES. Considering these findings, there is a pressing need to develop multi-source surveillance systems that integrate clinical, claims, and administrative data to more accurately identify GES. Standardized, statewide screening protocols must be adopted across hospitals to ensure consistency in GES identification and reporting. Moreover, integration of Medicaid claims and vital records systems could support cross-verification of GES, thereby enhancing public health response capabilities. Observed differences by hospital teaching/research status and size suggest that institutional practices and documentation workflows contribute to reporting gaps. Aligning clinical screening tools, diagnostic coding, and reporting requirements across facilities could improve consistency and equity in GES identification.

These findings underscore the need for increased public health investment to support maternal and child health programs, including expanded screening efforts for early detection of GES. More accurate identification of GES can inform more equitable, data-driven distribution of services and targeted investments in developmental screening, early childhood intervention and education services, and family-centered supports like home visiting programs. This is especially pertinent in rural and frontier communities and among populations experiencing higher discordance. In parallel, policies that reduce stigma, clarify mandatory reporting requirements, and promote non-punitive, trauma-informed approaches to prenatal substance use screening may improve patient disclosure and provider documentation, ultimately strengthening both surveillance and care delivery.

## 6. Conclusions

This study demonstrates substantial discordance in GES reporting between birth certificates and Medicaid claims, highlighting important limitations of relying on any single data source for population-level GES surveillance. By leveraging linked administrative datasets, we identified a higher burden of GES than would be captured through birth records alone and demonstrated that discordance varies systematically across patient demographics, geographic regions, and hospital characteristics. These findings underscore the value of linked administrative data for improving the accuracy and equity of GES surveillance efforts.

By highlighting key factors contributing to discordance, this study contributes new evidence on how institutional practices and population characteristics shape the completeness of GES documentation across data systems. In particular, the presence of hospital-level clustering suggests that institutional factors play a meaningful role in surveillance quality.

Finally, given the significant findings on overall discordance, further research is warranted to examine the directionality of discordance, with a particular focus on identifying predictors of underreporting on the birth certificate. Additionally, further research is needed to uncover the underlying factors contributing to the hospital-level clustering observed in this study. Continued refinement of multi-source surveillance approaches will be critical to improving the accuracy, equity, and public health utility of GES monitoring.

## Figures and Tables

**Table 3:** Multilevel model building process.

	Model 0	Model 1	Model 2	Model 3
Model building process				
Method	Null model with no random effect for the intercept	Null model with random effect for the intercept	Model 1 + patient-level predictors	Model 2 + hospital-level predictors
Model fit statistics				
−2 Res Log Likelihood	31,663.58	31,395.32	29,170.66	28,701.18
LRT ^		268.26	2224.66	469.48
AIC	31,665.58	31,399.32	29,206.66	28,749.18
BIC	31,674.41	31,401.5	29,226.3	28,774.24
Hospital clustering statistics				
ICC †		4.12%	1.18%	0.60%

^ Model 1 was compared to Model 0. Model 2 was compared to Model 1. Model 3 was compared to Model 2. † Formula for ICC: (random intercept variance)/(random intercept variance + 3.29), assuming standard logistic regression distribution.

**Table 4:** Discordance rates per 1000 Medicaid-covered live births by patient-level characteristics.

Patient-Level Characteristics	Annual Rate			Overall Rate
	(95% Confidence Intervals)			
	2022	2023	2024	(2022–2024)
Statewide	98.37	95.50	91.29	95.09
	(93.9–102.8)	(91.0–100.0)	(86.9–95.7)	(92.5–97.7)
Age				
<18	112.78	93.75	111.91	105.90
	(74.8–150.8)	(60.1–127.4)	(74.8–149.0)	(85.0–126.8)
18–29	95.92	95.43	89.46	93.67
	(90.3–101.6)	(89.6–101.3)	(83.8–95.2)	(90.4–97.0)
30–39	101.40	98.48	93.76	97.85
	(93.0–108.3)	(90.9–106.1)	(86.7–101.3)	(93.6–102.3)
40+	104.84	66.67	83.62	84.14
	(77.9–131.8)	(46.2–87.1)	(61.2–106.0)	(70.8–97.5)
Race/Ethnicity				
White non-Hispanic	167.53	152.65	149.52	156.87
	(156.56–178.5)	(141.7–163.6)	(138.6–160.4)	(150.5–163.2)
Black non-Hispanic	126.42	125.43	133.52	128.37
	(115.3–137.6)	(114.0–136.9)	(121.6–145.4)	(121.7–135.0)
Hispanic	50.90	53.88	48.07	50.92
	(46.1–55.7)	(48.9–58.9)	(43.4–52.7)	(48.1–53.7)
Other non-Hispanic	69.81	78.86	62.95	70.61
	(55.6–84.0)	(63.3–94.4)	(49.7–77.2)	(62.1–79.1)
Mother’s County of Residence				
Clark	85.04	84.12	84.07	84.42
	(80.4–89.7)	(79.4–88.9)	(79.4–88.8)	(81.7–87.1)
Washoe	141.85	127.32	101.39	123.22
	(126.5–157.2)	(112.9–141.7)	(88.4–114.4)	(115.0–131.5)
Rural/Frontier	173.35	163.07	148.61	161.75
	(152.9–193.8)	(142.6–183.5)	(129.2–168.0)	(150.1–173.4)
Educational Attainment				
Less than High School	116.61	115.89	112.98	115.20
	(106.0–127.2)	(104.9–126.9)	(102.1–123.8)	(109.0–121.4)
High School or GED	107.06	102.17	92.45	100.61
	(100.0–114.1)	(95.2–109.2)	(85.8–99.1)	(96.6–104.6)
Some College	80.86	81.86	80.82	81.17
	(72.8–88.9)	(73.3–90.4)	(72.3–89.4)	(76.3–86.0)
Bachelors Degree	39.06	26.18	41.04	35.73
	(27.2–50.9)	(16.1–36.3)	(29.2–52.9)	(29.1–42.3)
Masters or Higher	35.29	26.91	32.86	31.84
	(12.6–57.9)	(5.7–48.1)	(8.9–56.8)	(18.7–44.9)
Unknown	138.85	118.74	118.51	123.64
	(112.1–165.6)	(97.8–139.7)	(98.7–138.3)	(110.9–136.3)
Zip Code Median Household Income				
Quartile 1 (≤USD 50,945)	95.70	96.81	94.41	95.65
	(87.0–104.4)	(88.0–105.6)	(85.6–103.2)	(90.6–100.7)
Quartile 2 (USD 50,946–USD 65,798)	109.45	97.57	89.77	99.04
	(100.1–118.8)	(88.4–106.8)	(81.1–98.5)	(93.8–104.3)
Quartile 3 (USD 65,799–USD 85,801)	103.45	103.38	94.43	100.47
	(94.5–112.4)	(94.3–112.5)	(85.6–103.2)	(95.3–105.7)
Quartile 4 (≥USD 85,802)	83.07	82.75	86.20	84.01
	(74.6–91.5)	(74.1–91.4)	(77.5–94.9)	(79.0–89.0)

**Table 5:** Estimated odds ratios for multilevel logistic regression models by patient-level characteristics.

	Model 2		Model 3	
	Odds Ratio (95% Confidence Interval)	*p*-Value	Odds Ratio (95% Confidence Interval)	*p*-Value
Mother’s Age				
<18	0.850 (0.672–1.076)	0.1768	0.849 (0.670–1.076)	0.1749
18–29	reference		reference	
30–39	**1.122 (1.051–1.198)**	**0.0006**	**1.126 (1.054–1.203)**	**0.0004**
40+	0.980 (0.813–1.180)	0.8287	0.964 (0.798–1.164)	0.7028
Race/Ethnicity				
White non-Hispanic	reference		reference	
Black non-Hispanic	**0.868 (0.799–0.944)**	**0.0009**	**0.873 (0.802–0.949)**	**0.0015**
Hispanic	**0.267 (0.247–0.290)**	**<0.0001**	**0.268 (0.247–0.291)**	**<0.0001**
Other non-Hispanic	**0.472 (0.410–0.543)**	**<0.0001**	**0.475 (0.413–0.548)**	**<0.0001**
Mother’s County of Residence				
Clark	reference		reference	
Washoe	1.099 (0.887–1.363)	0.3883	1.231 (0.961–1.577)	0.0997
Rural/Frontier	**1.527 (1.299–1.796)**	**<0.0001**	**1.550 (1.299–1.851)**	**<0.0001**
Educational Attainment				
Less than Highschool	**1.3998 (1.289–1.516)**	**<0.0001**	**1.396 (1.286–1.514)**	**<0.0001**
High School or GED	reference		reference	
Some College	**0.775 (0.714–0.842)**	**<0.0001**	**0.774 (0.712–0.841)**	**<0.0001**
Bachelor’s Degree	**0.335 (0.176–0.409)**	**<0.0001**	**0.330 (0.269–0.404)**	**<0.0001**
Master’s or Higher	**0.273 (0.176–0.424)**	**<0.0001**	**0.278 (0.179–0.432)**	**<0.0001**
Unknown	1.002 (0.868–1.158)	0.9771	0.997 (0.863–1.152)	0.9674
Median Household Income				
Quartile 1 (≤USD 50,945)	**1.111 (1.015–1.216)**	**0.0228**	**1.097 (1.001–1.202)**	**0.0470**
Quartile 2 (USD 50,946–USD 65,798)	1.057 (0.968–1.153)	0.2150	1.043 (0.955–1.139)	0.3505
Quartile 3 (USD 65,799–USD 85,801)	reference		reference	
Quartile 4 (≥USD 85,802)	**0.802 (0.773–0.879)**	**<0.0001**	**0.797 (0.727–0.874)**	**<0.0001**

Note: Significant results are indicated by bold text.

**Table 6:** Estimated odds ratios for multilevel logistic regression models by hospital-level characteristics.

	Model 3	
	Odds Ratio (95% Confidence Interval)	*p*-Value
Hospital Type		
Public	0.806 (0.613–1.060)	0.1226
Private	reference	
Academic Status		
Non-teaching/research	reference	
Teaching/research	**1.355 (1.084–1.694)**	**0.0077**
Location		
Rural	0.902 (0.635–1.282)	0.5670
Urban	reference	
Bed Size		
Quartile 1 (≤60)	**1.695 (1.118–2.571)**	**0.0130**
Quartile 2 (61–199)	1.218 (0.978–1.517)	0.0778
Quartile 3 (200–393)	reference	
Quartile 4 (≥394)	1.042 (0.854–1.272)	0.6846

Note: Significant results are indicated by bold text.

## Data Availability

Restrictions apply to the availability of these data. Data were obtained from the Nevada Office of Analytics and are available from the authors with the permission of the Nevada Office of Analytics.

## Abbreviations

GES	Gestational Exposure to Substances
ICC	Intraclass Correlation Coefficient
CI	Confidence Interval
OR	Odds Ratio
NAS	Neonatal Abstinence Syndrome
NOWS	Neonatal Opioid Withdrawal Syndrome
IRB	Internal Review Board
OOS	Out of State
LRT	Likelihood Ratio Tests
AIC	Akaike Information Criterion
BIC	Bayesian Information Criterion
VIF	Variance Inflation Factors
NC	North Carolina
USA	United States of America

## Appendix A. International Classification of Diseases (ICD-10) Codes Used to Identify GES in Medicaid Claims

### Appendix A.1. Maternal Records

*Asterisks indicate that all subcodes with the corresponding leading substring were included.

Z72.0: Tobacco use
F10.*: Alcohol Related Disorders (excluding F10.11, F10.21, F10.91)
F11.*: Opioid Related Disorders (excluding F11.11, F11.21, F11.91)
F12.*: Cannabis Related Disorders (excluding F12.11, F12.21, F12.91)
F13.*: Sedative, hypnotic, or anxiolytic Related Disorders (excluding F13.11, F13.21, F13.91)
F14.*: Cocaine Related Disorders (excluding F14.11, F14.21, F14.91)
F15.*: Other Stimulant Related Disorders (excluding F15.11, F15.21, F15.91)
F16.*: Hallucinogen Related Disorders (excluding F16.11, F16.21, F16.91)
F17.*: Nicotine Related Disorders (excluding F17.11, F17.21, F17.91)
F18.*: Inhalant Related Disorders (excluding F18.11, F18.21, F18.91)
F19.*: Other Psychoactive Substance Related Disorders (excluding F19.11, F19.21, F19.91)
T40.0X1*: Poisoning by opium, accidental (unintentional)
T40.0X2*: Poisoning by opium, intentional self-harm
T40.0X3*: Poisoning by opium, assault
T40.0X4*: Poisoning by opium, undetermined
T40.0X5*: Adverse effect of opium
T40.1X1*: Poisoning by heroin, accidental (unintentional)
T40.1X2*: Poisoning by heroin, intentional self-harm
T40.1X3*: Poisoning by heroin, assault
T40.1X4*: Poisoning by heroin, undetermined
T40.1X5*: Adverse effect of heroin
T40.2X1*: Poisoning by other opioids, accidental (unintentional)
T40.2X2*: Poisoning by other opioids, intentional self-harm
T40.2X3*: Poisoning by other opioids, assault
T40.2X4*: Poisoning by other opioids, undetermined
T40.2X5*: Adverse effect of other opioids
T40.3X1*: Poisoning by methadone, accidental (unintentional)
T40.3X2*: Poisoning by methadone, intentional self-harm
T40.3X3*: Poisoning by methadone, assault
T40.3X4*: Poisoning by methadone, undetermined
T40.3X5*: Adverse effect of methadone
T40.411*: Poisoning by fentanyl or fentanyl analogs, accidental (unintentional)
T40.412*: Poisoning by fentanyl or fentanyl analogs, intentional self-harm
T40.413*: Poisoning by or fentanyl analogs, assault
T40.414*: Poisoning by other fentanyl or fentanyl analogs, undetermined
T40.415*: Adverse effect of fentanyl or fentanyl analogs
T40.421*: Poisoning by tramadol, accidental (unintentional)
T40.422*: Poisoning by tramadol, intentional self-harm
T40.423*: Poisoning by tramadol, assault
T40.424*: Poisoning by tramadol, undetermined
T40.425*: Adverse effect of tramadol
T40.421*: Poisoning by other synthetic narcotics, accidental (unintentional)
T40.422*: Poisoning by other synthetic narcotics, intentional self-harm
T40.423*: Poisoning by other synthetic narcotics, assault
T40.424*: Poisoning by other synthetic narcotics, undetermined
T40.425*: Adverse effect of other synthetic narcotics
T40.5X1*: Poisoning by cocaine, accidental (unintentional)
T40.5X2*: Poisoning by cocaine, intentional self-harm
T40.5X3*: Poisoning by cocaine, assault
T40.5X4*: Poisoning by cocaine, undetermined
T40.5X5*: Adverse effect of cocaine
T40.601*: Poisoning by unspecified narcotics, accidental (unintentional)
T40. 602*: Poisoning by unspecified narcotics, intentional self-harm
T40. 603*: Poisoning by unspecified narcotics, assault
T40. 604*: Poisoning by unspecified narcotics, undetermined
T40. 605*: Adverse effect of unspecified narcotics
T40.691*: Poisoning by other narcotics, accidental (unintentional)
T40. 692*: Poisoning by other narcotics, intentional self-harm
T40. 693*: Poisoning by other narcotics, assault
T40. 694*: Poisoning by other narcotics, undetermined
T40. 695*: Adverse effect of other narcotics
T40.711*: Poisoning by cannabis, accidental (unintentional)
T40. 712*: Poisoning by cannabis, intentional self-harm
T40. 713*: Poisoning by cannabis, assault
T40. 714*: Poisoning by cannabis, undetermined
T40. 715*: Adverse effect of cannabis
T40.721*: Poisoning by synthetic cannabinoids, accidental (unintentional)
T40. 722*: Poisoning by synthetic cannabinoids, intentional self-harm
T40. 723*: Poisoning by synthetic cannabinoids, assault
T40. 724*: Poisoning by synthetic cannabinoids, undetermined
T40. 725*: Adverse effect of synthetic cannabinoids
T40.8X1*: Poisoning by lysergide [LSD], accidental (unintentional)
T40. 8X2*: Poisoning by lysergide [LSD], intentional self-harm
T40. 8X3*: Poisoning by lysergide [LSD], assault
T40. 8X4*: Poisoning by lysergide [LSD], undetermined
T40.901*: Poisoning by unspecified psychodysleptics [hallucinogens], accidental (unintentional)
T40. 902*: Poisoning by unspecified psychodysleptics [hallucinogens], intentional self-harm
T40. 903*: Poisoning by unspecified psychodysleptics [hallucinogens], assault
T40. 904*: Poisoning by unspecified psychodysleptics [hallucinogens], undetermined
T40. 905*: Adverse effect of unspecified psychodysleptics [hallucinogens]
T40.991*: Poisoning by other psychodysleptics [hallucinogens], accidental (unintentional)
T40. 992*: Poisoning by other psychodysleptics [hallucinogens], intentional self-harm
T40. 993*: Poisoning by other psychodysleptics [hallucinogens], assault
T40. 994*: Poisoning by other psychodysleptics [hallucinogens], undetermined
T40. 995*: Adverse effect of other psychodysleptics [hallucinogens]
T51: Toxic effect of alcohol
O99.32*: Drug use complicating pregnancy, childbirth, and the puerperium
O99.31*: Alcohol use complicating pregnancy, childbirth, and the puerperium

### Appendix A.2. Infant Records

P96.1: Neonatal withdrawal symptoms from maternal use of drugs of addiction
P04.2: Newborn affected by maternal use of tobacco
P04.3: Newborn affected by maternal use of alcohol
Q86.0: Fetal alcohol syndrome (dysmorphic)
P04.40: Newborn affected by maternal use of unspecified drugs of addiction
P04.41: Newborn affected by maternal use of cocaine
P04.42: Newborn affected by maternal use of hallucinogens
P04.49: Newborn affected by maternal use of other drugs of addiction
P04.14: Newborn affected by maternal use of opioids
P04.16: Newborn affected by maternal use of amphetamines
P04.81: Newborn affected by maternal use of cannabis
